# Association between Surgical Waiting Time and Postoperative Recurrence Risk in Clinical T1bN0M0 Thoracic Esophageal Squamous Cell Carcinoma: A Multicenter Retrospective Observational Study

**DOI:** 10.1245/s10434-026-19840-6

**Published:** 2026-06-05

**Authors:** Yutaka Miyawaki, Hisashi Fujiwara, Yuki Shiko, Shigeo Haruki, Youichi Kumagai, Akio Kaito, Yasuaki Nakajima, Kazuya Yamaguchi, Hiroshi Sato, Shinichi Sakuramoto

**Affiliations:** 1 Department of Gastroenterological Surgery, Saitama Medical University International Medical Center, Hidaka-shi, Saitama Japan; 2 Department of Gastroenterological Surgery, Institute of Science Tokyo, Tokyo, Japan; 3 Department of Biostatistics, Graduate School of Medicine, Saitama Medical University, Saitama, Japan; 4 Department of Esophageal Surgery, Tokyo Metropolitan Cancer and Infectious Diseases Center Komagome Hospital, Tokyo, Japan; 5 Department of Digestive Tract and General Surgery, Saitama Medical Center, Saitama Medical University, Saitama, Japan; 6 Department of Surgery, Tsuchiura Kyodo General Hospital, Ibaraki, Japan; 7 Department of Surgery, Edogawa Hospital, Tokyo, Japan; 8 Department of Surgery, Musashino Red Cross Hospital, Tokyo, Japan

**Keywords:** Esophageal cancer, Esophagectomy, Local neoplasm recurrence, Neoplastic processes, Treatment delay

## Abstract

**Background:**

Although prompt treatment is desirable for malignancies, surgical delays are sometimes unavoidable. Previous studies show conflicting results on the effect of diagnosis-to-surgery delay in esophageal cancer, mainly focusing on advanced stages treated preoperatively. Early stage disease, particularly cT1bN0, often involves longer waiting times, but the acceptable delay remains unclear. We conducted this study to evaluate the impact of surgical waiting time on postoperative survival in patients with clinical T1bN0M0 esophageal squamous cell carcinoma (ESCC) undergoing upfront esophagectomy.

**Patients and Methods:**

This multicenter retrospective study included 160 patients with cT1bN0M0 ESCC undergoing subtotal esophagectomy with lymphadenectomy at seven Japanese institutions between 2008 and 2021. Receiver operating characteristic (ROC) analysis identified the optimal waiting time cutoff predicting recurrence. Survival outcomes were compared between short and long waiting groups using the Kaplan–Meier method and Cox regression analyses.

**Results:**

ROC analysis identified 66.5 days as the optimal cutoff value. The long waiting group (≥ 67 days) showed significantly worse 3-year recurrence-free survival (87.9% versus 73.5%, log-rank* P* < 0.01; hazard ratio 2.71, 95% confidence interval 1.29–5.70, *P* < 0.01), whereas cancer-specific survival showed no significant difference. Multivariate analysis identified prolonged waiting time, pathological T2 or deeper invasion, and lymph node metastasis as independent predictors of recurrence, with longer delays linked to increased systemic recurrence beyond 2 years postoperatively.

**Conclusions:**

In patients with cT1bN0 ESCC, surgical delay beyond approximately 2 months independently increases postoperative recurrence. It is suggested that planning surgery without delay reduces the risk of recurrence; however, verification in a larger cohort is necessary.

**Supplementary Information:**

The online version contains supplementary material available at https://doi.org/10.1245/s10434-026-19840-6.

Malignant tumors, including esophageal cancer, are expected to progress over time; therefore, prompt treatment is desirable. Esophagectomy remains the standard therapy for locally advanced esophageal cancer.^1–4^ However, treatment delays are sometimes unavoidable, raising concern for worse outcomes because esophageal cancer is an aggressive disease that metastasizes to lymph nodes early.^5^ In cT1bN0 esophageal cancer, typically managed with upfront surgery, the lower oncologic urgency compared with more advanced stages with clinically evident metastases may result in longer surgical waiting times. In addition, the increasing use of endoscopic resection for superficial esophageal carcinoma has inevitably prolonged waiting time for surgery in cases requiring additional treatment based on postresection pathology.

Although some studies suggest that prolonged surgical waiting time is associated with poorer survival outcomes,^6,7^ others report no significant effect of diagnostic-to-surgical delay on long-term survival,^8,9^ leaving the impact unclear. Furthermore, most previous studies have focused on advanced disease treated with preoperative therapy, whereas few have examined early esophageal cancer, which is more likely to involve prolonged surgical waiting time. Notably, no study appears to have specifically focused on esophageal squamous cell carcinoma (ESCC).

Therefore, determining an acceptable surgical waiting time for early ESCC that prevents clinical tumor progression according to the TNM classification while maintaining acceptable postoperative survival is critical. Accordingly, we investigated the impact of waiting time from diagnosis to surgery on long-term survival in patients with clinical T1bN0 ESCC using data from a retrospective multicenter cohort.

## Patients and Methods

### Patients and Study Design

This multicenter study was conducted across seven institutions in Japan, including four high-volume centers and three city hospitals. A CONSORT diagram is shown in Supplementary Fig. 1. We retrospectively reviewed patients with cT1bN0M0 thoracic ESCC who underwent upfront subtotal esophagectomy with lymph node dissection between 2008 and 2021. Patients with fewer than 10 dissected lymph nodes, censoring within 180 days, or receipt of postoperative adjuvant therapy were excluded. Ultimately, 160 eligible cases were included. A total of 49 patients had surgical waiting times exceeding 60 days. Most patients underwent tumor re-evaluation before surgery, and no tumor progression from cT1bN0 was observed. Only four patients had intervals exceeding 60 days between the final tumor evaluation and surgery, ranging from 62 to 83 days.

Patient characteristics, clinicopathological factors, history of endoscopic resection, surgical procedures, and prognoses were reviewed. Surgical waiting time was defined as the period from the first visit to the date of surgery for patients diagnosed with ESCC at another hospital and referred to the study institution, or from the date of diagnosis to surgery for those incidentally diagnosed during routine visits for other diseases. The clinical stage was determined using esophagogastroduodenoscopy and computed tomography (CT) before treatment. Preoperative lymph node metastasis diagnosis was primarily assessed using CT. For lymph nodes with a short-axis diameter of ≥ 5 mm, metastasis was diagnosed on the basis of a comprehensive assessment of location and morphology. Regarding positron emission tomography (PET)-CT, it was routinely performed at two institutions, whereas the remaining five institutions performed it on a case-by-case basis. Overall, PET-CT was performed in 96 cases (60.0%). The extent of tumor spread was evaluated using the eighth edition of the TNM classification established by the Union for International Cancer Control. This study was approved by the Ethics Committee of Saitama Medical University International Medical Center and all participating institutions.

### Treatment and Postoperative Follow-Up

Esophagectomy and lymph node dissection were performed according to the Japanese Esophageal Cancer Treatment Guidelines.^3^ Postoperative follow-up is standard clinical practice in Japan and includes esophagogastroduodenoscopy and CT every 4–6 months for the first year after surgery. Recurrence-free survival (RFS) was calculated from the date of the surgery to the date of postoperative recurrence. Cancer-specific survival (CSS) was calculated from the date of surgery to the date of death attributable to ESCC. Noncancer-related deaths were treated as censored cases on the date of death in the CSS analysis.

### Statistical Analysis

Receiver operating characteristic (ROC) curve analysis was performed to identify the optimal surgical waiting time cutoff value for predicting postoperative recurrence or cancer-specific death. The optimal cutoff, determined as the point maximizing Youden’s index (sensitivity + specificity −1), was then used to divide patients into short and long waiting time groups. Baseline characteristics were subsequently summarized for each group. Continuous variables are presented as the mean ± standard deviation, and categorical variables as numbers (percentages). Comparisons between groups were made using the *χ*^2^ test or Fisher’s exact test for categorical variables and the Mann–Whitney *U* test for continuous variables, as appropriate. For comparisons among three or more groups, one-way analysis of variance was performed, and pairwise comparisons between groups were adjusted using the Bonferroni method. RFS and CSS were estimated using the Kaplan–Meier method, and differences between groups were compared using the log-rank test. For RFS, a sensitivity analysis was also performed by comparing groups using alternative cutoff values of 1, 2, and 3 months for surgical waiting time. Hazard ratios (HRs) for univariate and multivariate survival analyses were calculated using Cox proportional hazards models. Multivariate analysis included surgical waiting time along with conventional prognostic factors: pathological T and N factors and lympho-vascular invasion as variables. Multicollinearity between the covariates used in multivariate analysis and the waiting time for surgery was verified using the coefficient of variance inflation factor (VIF). Statistical analyses were performed using SPSS software (version 30.0; IBM Corp., Armonk, NY, USA). Two-sided tests were applied, and *P*-values < 0.05 were considered statistically significant.

## Results

### Surgical Waiting Time

**Fig. 1:**
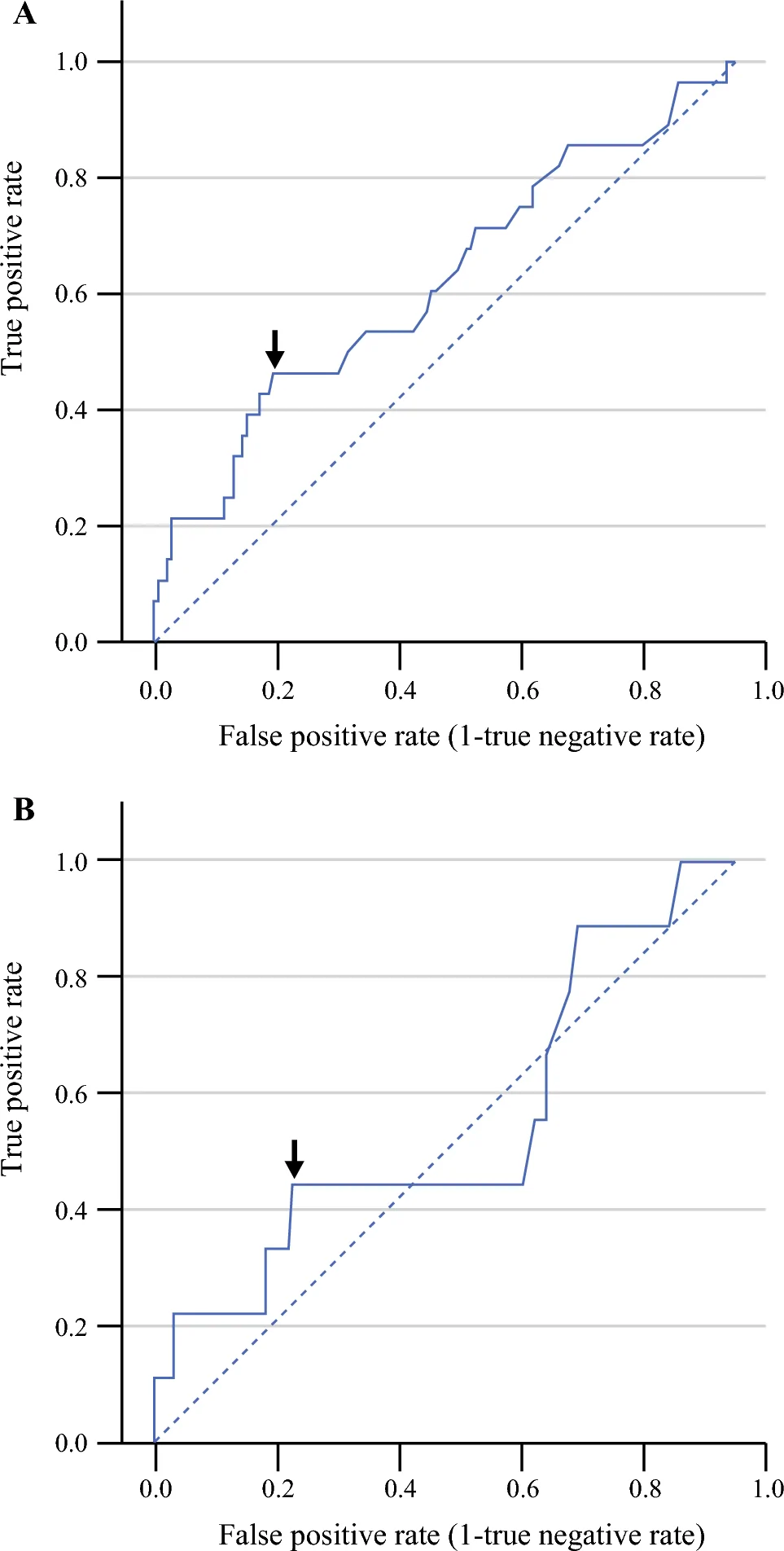
**A** Receiver operating characteristic curve using surgical waiting time to predict postoperative recurrence, with an area under the curve of 0.62, sensitivity of 46.4%, and specificity of 79.5%; the diagonal dotted line indicates the reference line and the black arrow indicates the cutoff of 66.5 days according to Youden’s index; **B** receiver operating characteristic curve using surgical waiting time to predict cancer-specific survival, with an area under the curve of 0.55, a sensitivity of 44.4%, and a specificity of 76.2%; the dotted diagonal line represents the reference and the black arrow indicates a cutoff of 66.5 days according to Youden’s index

Supplementary Fig. 2 shows the surgical waiting times. The median time to surgery was 47.5 days (interquartile range 34.25–66.75 days). ROC curve analysis for predicting postoperative recurrence revealed an area under the curve of 0.62 (95% confidence interval [CI] 0.50–0.74). Using the optimal cutoff value of 66.5 days for surgical waiting time, determined according to Youden’s index, the sensitivity was 46.4%, and the specificity was 79.5% (Fig. 1A). On the basis of this cutoff, 120 patients were assigned to the short waiting time group and 40 patients to the long waiting time group. Likewise, ROC curve analysis for predicting cancer-specific death revealed an area under the curve of 0.55 (95% CI 0.33–0.76). The optimal cutoff value for the surgical waiting time for cancer-specific death was determined to be 66.5 days, the same as that for postoperative recurrence, with a sensitivity of 44.4% and a specificity of 76.2% (Fig. 1B).

### Patient Characteristics

Table 1 presents the clinical, surgical, and pathological characteristics of the short and long waiting time groups. No significant difference was observed in the change in the proportions of these two groups across the three periods from 2008 to 2021. There were 20 patients (12.5%) who underwent surgery as an additional treatment following pathological findings before endoscopic submucosal dissection (ESD), three in the short waiting time group and 17 in the long waiting time group. Therefore, the proportion of patients who had a prior ESD was significantly higher in the long waiting time group. In Japan, supraclavicular lymph nodes are treated as regional lymph nodes, and supraclavicular lymph node dissection was performed in 65 cases (40.6%). Pathological supraclavicular lymph node metastasis was observed in five of these cases, with a significantly higher rate in the long waiting time group. No significant differences were observed in other factors between the two groups.

**Table 1:** Comparison of patient characteristics between short and long waiting time for surgery groups

	All Patients	Waiting time < 66.5 days n=120	Waiting time > 66.5 days n=40	*p* value
Waiting time, days				
Mean (SD)	56.5 (36.6)	40.5 (12.5)	104.3 (43.0)	
Period				
2008-2012 (%)	22	16 (72.7)	6 (27.3)	0.90
2013-2017 (%)	81	62 (76.5)	19 (23.5)	
2017-2021 (%)	57	42 (73.7)	15 (26.3)	
Age, years				
Mean (SD)	68.5 (7.5)	68.2 (7.7)	69.5 (6.7)	0.37
Sex				
Female (%)	27	21 (77.8)	6 (22.2)	0.81
Male (%)	133	99 (74.4)	34 (25.6)	
ECOG performance status				
0 (%)	111	83 (74.8)	28 (25.2)	0.84
1–3 (%)	41	30 (73.2)	11 (26.8)	
(Unknown)	(8)			
Institution type				
High-volume center (%)	130	94 (72.3)	36 (27.7)	0.16
City hospital (%)	30	26 (86.7)	4 (13.3)	
Tumor Location				
Ut (%)	24	17 (70.8)	7 (29.2)	0.46
Mt (%)	92	67 (72.8)	25 (27.2)	
Lt (%)	44	36 (81.8)	8 (18.2)	
History of ESD treatment				
Absence (%)	140	117 (83.6)	23 (16.4)	< 0.01*
Presence (%)	20	3 (15.0)	17 (85.0)	
Tumor length diameter, mm				
Mean (SD)	36.1 (21.3)	35.6 (20.1)	37.8 (22.5)	0.46
Lymphovascular invasion				
Absence (%)	72	59 (81.9)	13 (18.1)	0.07
Presence (%)	88	61 (69.3)	27 (30.7)	
pT				
T1a (%)	42	34 (81.0)	8 (19.0)	0.58
T1b (%)	110	80(72.7)	30 (27.3)	
T2–3 (%)	8	6 (75.0)	2 (25.0)	
pN				
pN0 (%)	114	84 (73.7)	30 (26.3)	0.56
pN1 (%)	37	30 (81.8)	7 (18.9)	
pN2–3 (%)	9	6 (66.7)	3 (33.3)	
pM				
pM0 (%)	60	44 (73.3)	16 (31.7)	0.03*
pM1 (%)	5	1 (20.0)	4 (80.0)	
(Unknown)	(95)			
Thoracic procedure				
Open thoracotomy (%)	42	31 (73.8)	11 (26.2)	0.62
MIE (%)	107	82 (76.6)	25 (23.4)	
Mediastinoscopic surgery (%)	11	7 (63.6)	4 (36.4)	
Field of lymph node dissection				
Two field (%)	96	75 (78.1)	21 (21.9)	0.27
Three field (%)	64	45 (70.3)	19 (29.7)	
Reconstruction route				
Retrosternal (%)	129	100 (77.5)	29 (22.5)	0.32
Mediastinal (%)	19	12 (63.2)	7 (36.8)	
Antethoracic (%)	12	8 (66.7)	4 (33.3)	
Reconstruction organ				
Gastric tube (%)	155	118 (76.1)	37 (23.9)	0.11
Jejunum (%)	1	0 (0.0)	1 (100.0)	
Colon (%)	4	2 (50.0)	2 (50.0)	
Operation time, min				
Mean (SD)	465.2 (101.4)	464.1 (91.9)	468.3 (126.8)	0.81
Intraoperative blood loss, ml				
Mean (SD)	419.4 (484.6)	405.3 (457.4)	462.0 (563.4)	0.76
Number of lymph nodes dissected				
Mean (SD)	34.8 (15.8)	35.1 (16.0)	34.0 (15.3)	0.67

The percentage in parentheses indicates the row percentage. Unknown cases in pM did not undergo neck lymph node dissection. SD: standard deviation, ESD: endoscopic mucosal resection, * p>0.05

### Long-Term Survival

**Fig. 2:**
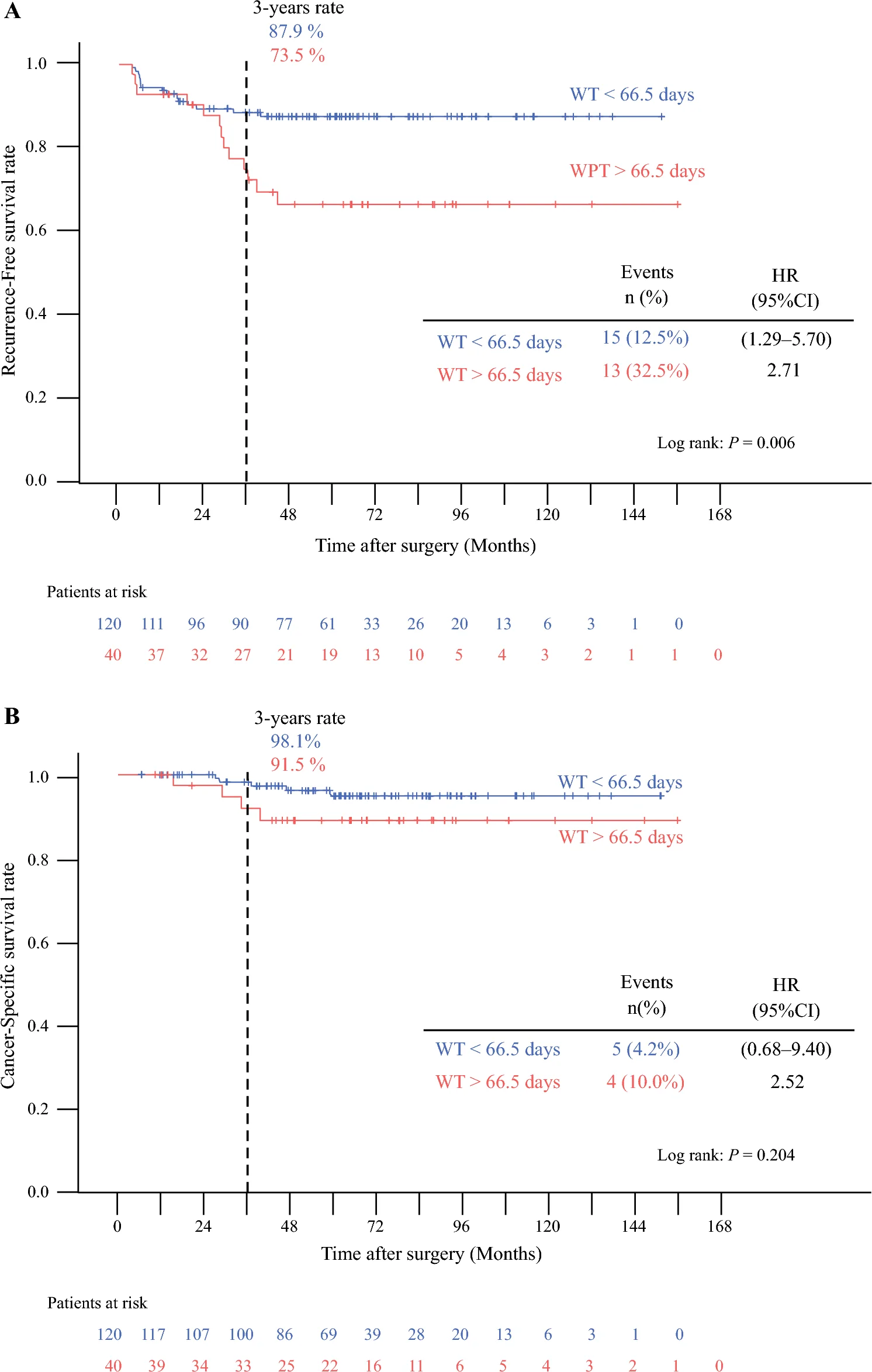
**A** Kaplan–Meier curves for recurrence-free survival (RFS); the long waiting time (> 66.5 days) group showed significantly worse RFS than the short waiting time (< 66.5 days) group (3-year RFS short/long: 87.9%/73.5%, log-rank* P* < 0.01); the hazard ratio (HR) was 2.71 (95% CI 1.29–5.70) in the long waiting time group, with the short waiting time group as the reference; **B** Kaplan–Meier curves of cancer-specific survival (CSS); no significant differences were observed between the two groups in terms of CSS (3-year CSS short/long: 98.1%/91.5%, log-rank* P* = 0.15); HR was 2.52 (95% CI 0.68–9.40) in the long waiting time group, with the short waiting time group as the reference

**Fig. 3:**
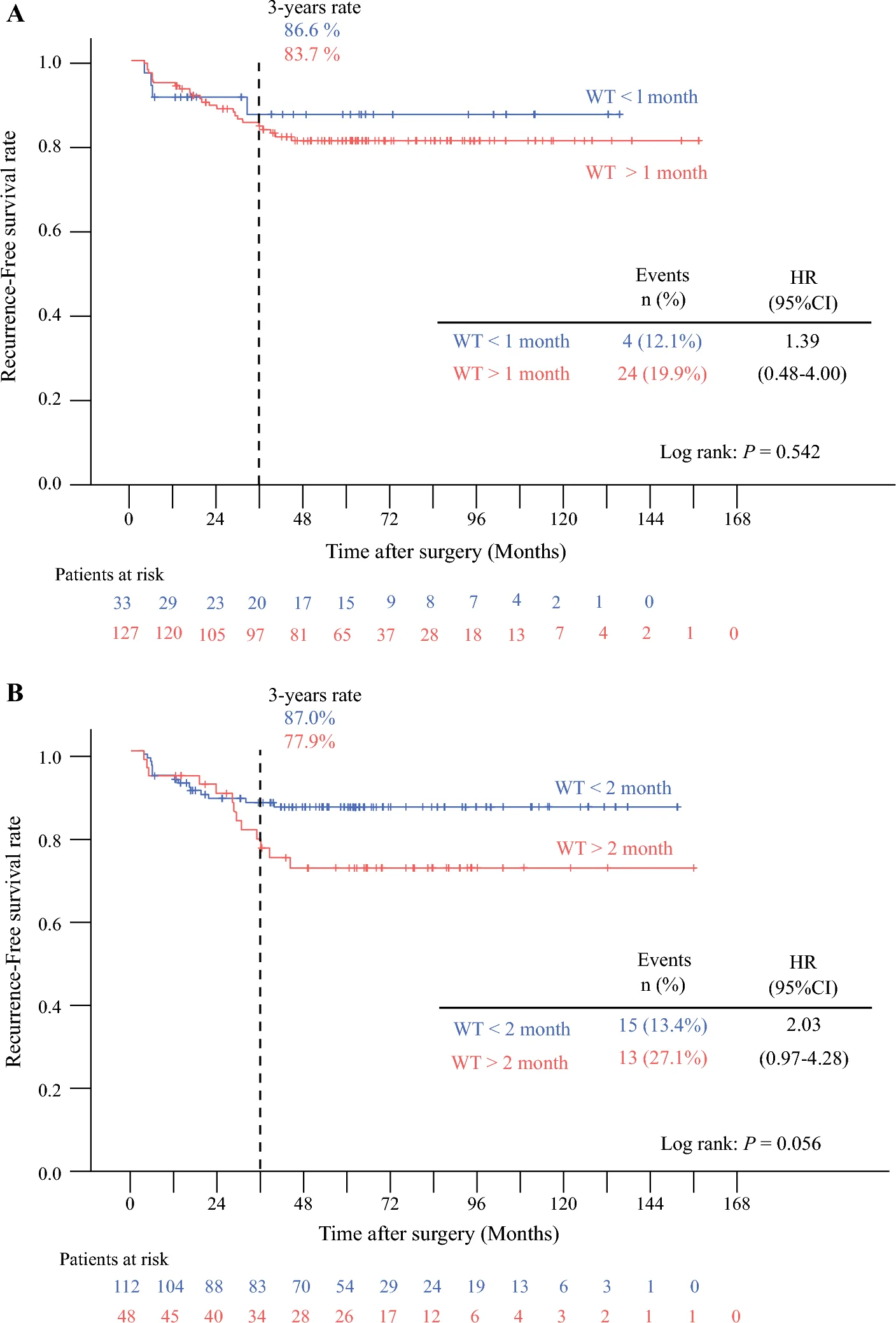

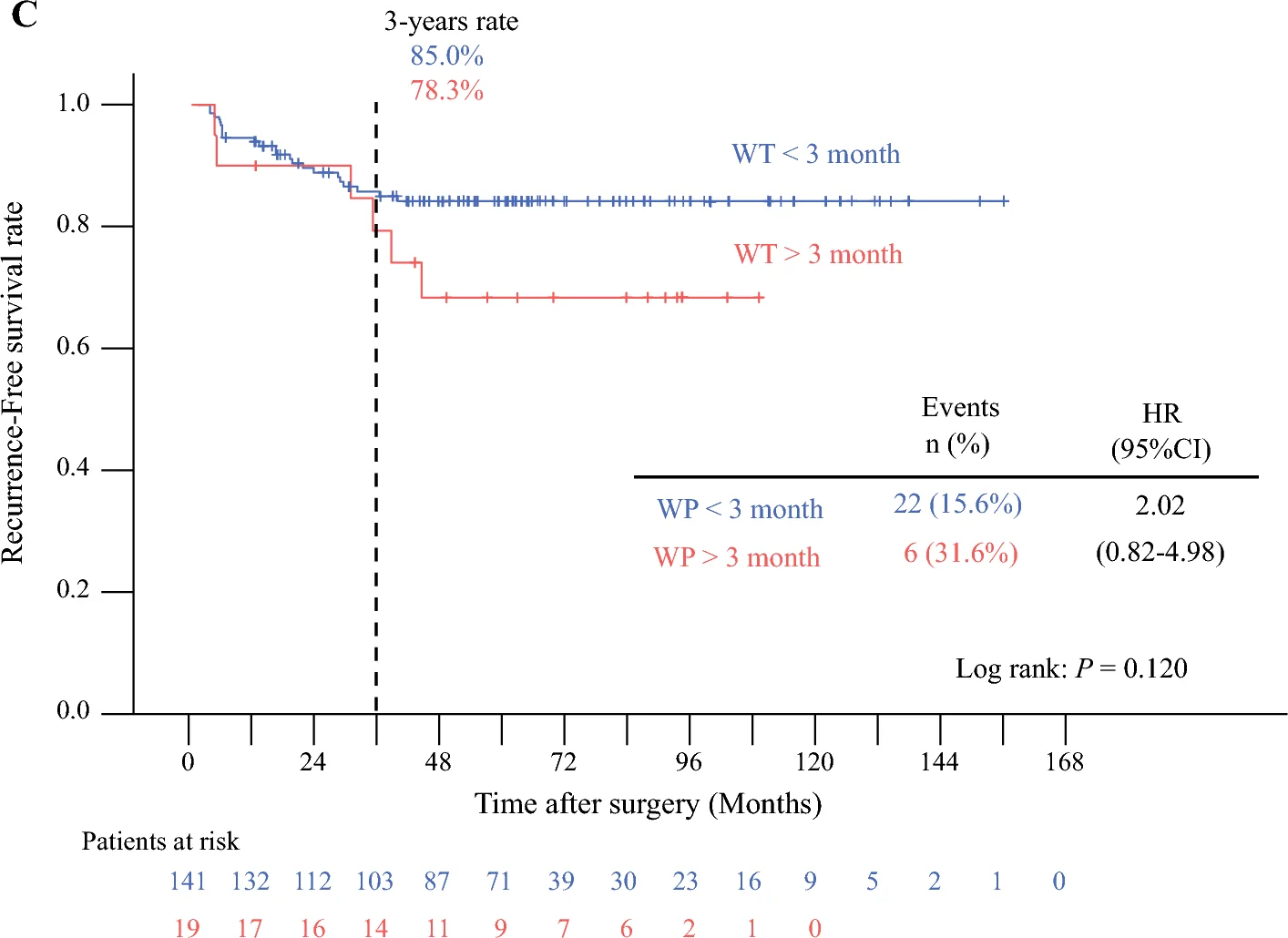
**A** Kaplan–Meier curve for recurrence-free survival (RFS) at a surgical waiting time cutoff of 1 month. No significant differences were observed between the two groups in terms of RFS (3-year RFS short/long, 86.6%/83.7%); HR was 1.39 (95% CI 0.48–4.00) in the long waiting time group, with the short waiting time group as the reference; **B** Kaplan–Meier curve for RFS at a surgical waiting time cutoff value of 2 months; no significant differences were observed between the two groups in terms of RFS (3-year RFS short/long, 87.0%/77.9%); HR was 2.03 (95% CI 0.97–4.28) in the long waiting time group, with the short waiting time group as the reference; **C** Kaplan–Meier curve for RFS at a surgical waiting time cutoff of 3 months; no significant differences were observed between the two groups in terms of RFS (3-year RFS short/long, 85.0%/78.3%); HR was 2.02 (95% CI 0.82–4.98) in the long waiting time group, with the short waiting time group as the reference

The median follow-up period of survivors was 61.1 months. The 3-year RFS rates were 84.2% (95% CI 81.2–87.2%), and the 3-year CSS rates were 96.5% (95% CI 94.9–99.1%), respectively. Figure 2 shows the Kaplan–Meier RFS and CSS estimates of survival in the short and long waiting time groups. The long waiting time group showed significantly worse RFS than the short waiting time group (3-year RFS short/long: 87.9%/73.5%, log-rank* P* < 0.01). The HR was 2.71 (95% CI 1.29–5.70, *P* < 0.01) in the long waiting time group, with the short waiting time group as the reference. However, no significant differences were observed between the two groups in terms of CSS (3-year CSS short/long: 98.1%/91.5%, log-rank* P* = 0.15). The HR was 2.52 (95% CI 0.68–9.40, *P* = 0.17) in the long waiting time group, with the short waiting time group as the reference. In the survival analysis of RFS in groups with cutoffs of 1, 2, and 3 months, the 3-year RFS was worse in the long waiting time group than in the short waiting time group; however, the difference was not statistically significant (Fig. 3). Table 2 presents the associations between clinical and pathological factors and RFS in univariate and multivariate Cox proportional hazards regression analysis. In univariate analysis, pathological tumor depth of T2 or deeper (HR 4.48; 95% CI 1.55–13.0; *P* < 0.01), pathological lymph node metastasis (HR 2.15; 95% CI 1.02–4.55; *P* = 0.05), lymphovascular invasion (HR, 2.78; 95% CI 1.18–6.55; *P* = 0.02), and a surgical waiting time of 67 days or longer were associated with RFS. Multivariate analysis using these factors showed that pathological tumor depth of T2 or deeper (HR 4.48; 95% CI 1.54–13.0; *P* < 0.01), pathological lymph node metastasis (HR 2.22; 95% CI 1.05-4.71; *P* = 0.04), and surgical waiting time of 67 days or longer (HR 2.86; 95% CI 1.36–6.04; *P* < 0.01) were independent prognostic factors. Multicollinearity between surgical waiting time and pathological T2 or deeper, pathological lymph node metastasis, and lymphovascular invasion was verified using VIF, and no multicollinearity was found (VIF = 1.02, 1.08, and 1.10, respectively).

**Table 2:** Univariate and multivariate analyses of prognostic factors for recurrence-free survival

Factor	Category	Recurrence-free survival					
		Univariate			Multivariate		
		HR	95% CI	*p* value	HR	95% CI	*p* value
Waiting time	≥67 days (vs. <67 days)	2.71	1.29–5.70	< 0.01 *	2.86	1.36-6.04	< 0.01 *
Age	≥75 (vs. <75)	0.65	0.25–1.71	0.38			
Sex	Female (vs. Male)	2.05	0.90–4.66	0.09			
ECOG performance status	1-3 (vs. 0)	0.89	0.38–2.13	0.80			
Institution type	City hospital (vs. High-volume center)	0.87	0.33–2.29	0.78			
Tumor location	Mt or Lt (vs. Ut)	0.78	0.30–2.05	0.61			
History of ESD treatment	presence (vs. absence)	1.10	0.38–3.18	0.86			
pT factor	pT2-3 (vs. T1a-1b)	4.48	1.55–13.0	< 0.01*	4.48	1.54–13.0	< 0.01 *
pN factor	pN1-3 (vs. N0)	2.15	1.02–4.55	0.05*	2.22	1.05–4.71	0.04 *
pM factor	pM1 (vs. M0)	1.25	0.16–9.76	0.83			
Tumor length diameter	≥32.5mm (vs. <32.5mm)	1.35	0.64–2.85	0.43			
Lymphovascular invasion	presence (vs. absence)	2.78	1.18–6.55	0.02*	1.71	0.68–4.33	0.26
Thoracic procedure	Open thoracomy (vs. MIE or MeE)	1.13	0.48–2.65	0.79			
Field of lymph node dissection	3Field (vs. 2Field)	1.00	0.47–2.12	0.99			
Operation time	≥460 min (vs. <460 min)	1.35	0.64–2.85	0.44			
Intraoperative blood loss	≥250 ml (vs. <250 ml)	0.91	0.43–1.93	0.80			

ESD: Endscopic submucosal deisectomy, HR: hazard ratio, CI: confidence interval, Ut: Upper thoracic, Mt: Middle thoracic, Lt: Lower thoracic, SCC: squamous cell carcinoma, MIE: Minimally invasive esophagectomy, MeE: Mediastinoscopic Esophagectomy, *p > 0.05

### Association between Surgical Waiting Time and RFS in Stratified Groups by Prior ESD

Supplementary Fig. 3 shows the Kaplan–Meier RFS estimates of survival in the short and long waiting time groups in subgroup analyses of 140 cases without prior ESD and 20 cases after prior ESD. In the subgroup without prior ESD, consistent with the overall population, the long waiting time group showed significantly worse RFS than the short waiting time group (3-year RFS short/long: 87.6%/72.3%, log-rank* P* < 0.01). Although the three cases in the short waiting time group after prior ESD had all progressed without recurrence, there was no significant difference between the two groups in post-ESD subgroup (3-year RFS short/long: 100%/75.3%, log-rank* P* = 0.37).

### Correlation between Surgical Waiting Time and Pathological Tumor Depth and Lymph Node Metastasis

**Fig. 4:**
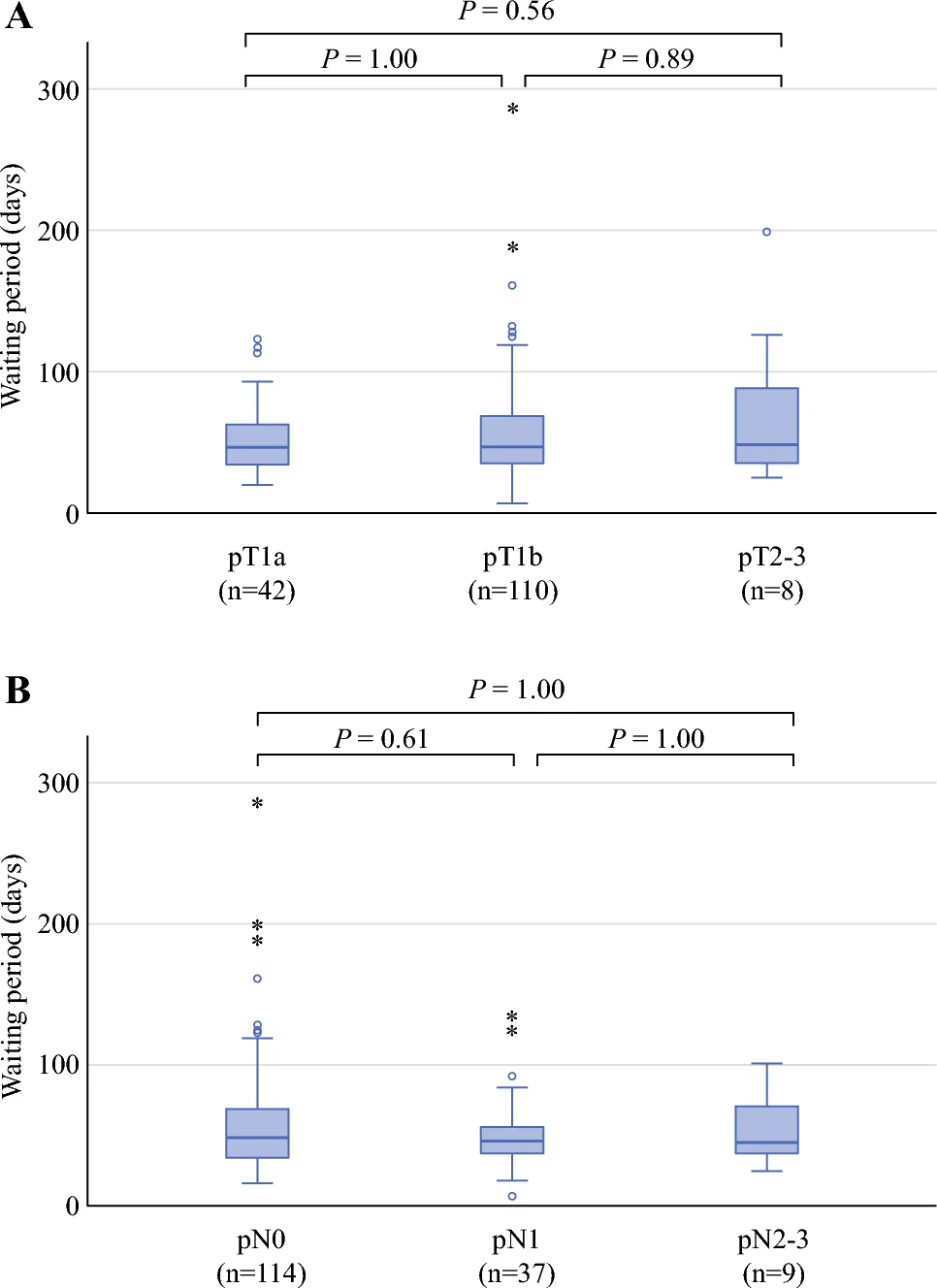
**A** Analysis of the correlation between pT1a, pT1b, pT2–3, and surgical waiting time; no difference in surgical waiting time was observed between the tumor depth groups; **B** analysis of the correlation between pN0, pN1, and pN2, and surgical waiting time; no difference in surgical waiting time was observed between the lymph node metastasis categories

In the analysis of the correlation between pT1a, pT1b, pT2-3, and surgical waiting time, no difference in surgical waiting time was observed in the pairwise comparisons between each tumor depth group (Fig. 4A). Similarly, in the analysis of the correlation between pN0, pN1, pN2, and surgical waiting time, no difference in surgical waiting time was observed in the pairwise comparisons between each lymph node metastasis category (Fig. 4B).

### Correlation between Surgical Waiting Time, Postoperative Recurrence Pattern, and Time to Recurrence

**Fig. 5:**
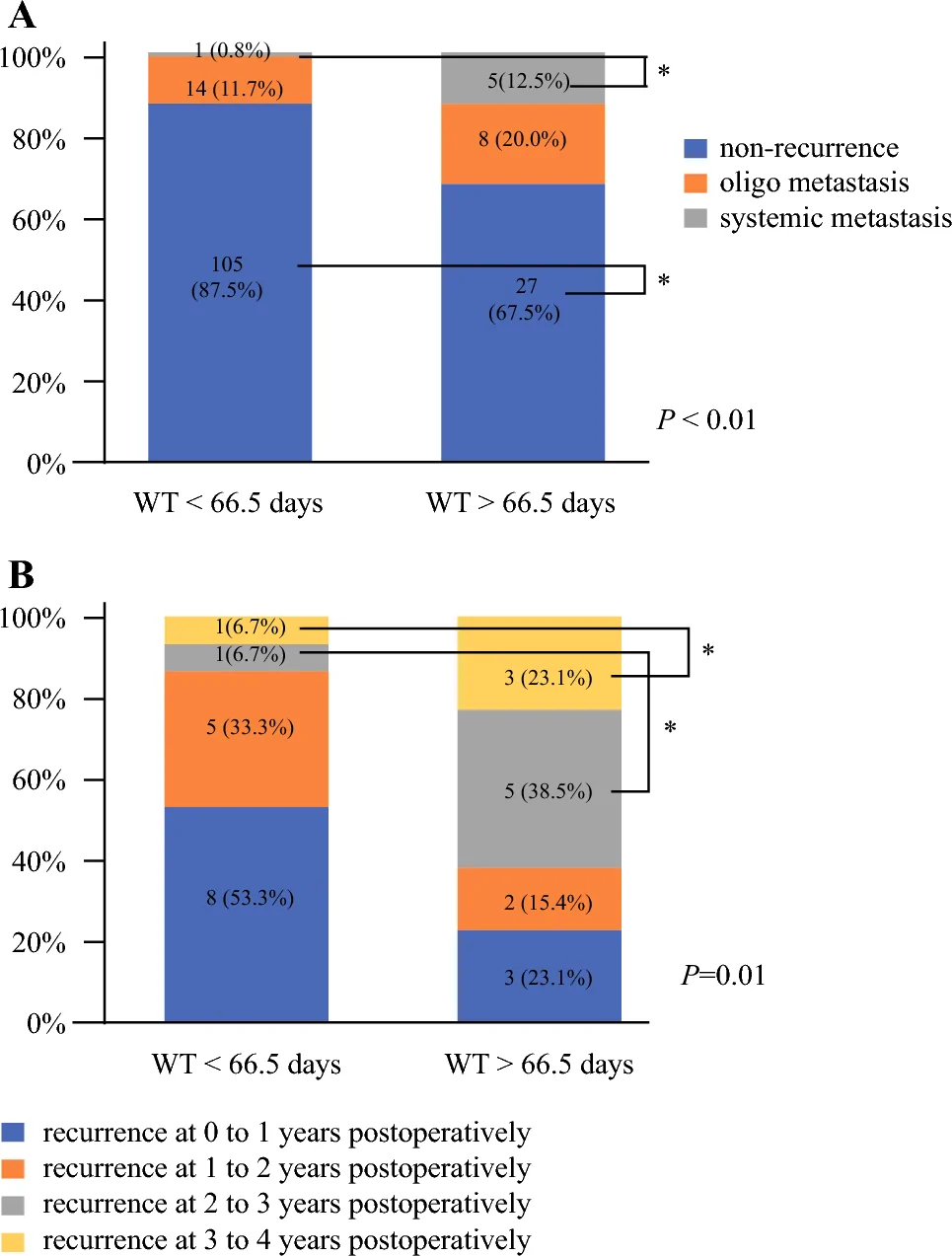
**A** Comparison of the incidence rates of postoperative recurrence patterns for nonrecurrence, oligometastasis, and systemic recurrence between the two groups with short and long waiting times for surgery; a significant difference was observed between the two groups, with a significantly higher incidence of systemic recurrence in the long waiting time group; asterisks indicate significant differences in intergroup comparisons; **B** a comparison of the percentage of postoperative recurrence periods divided into 1-year intervals between groups with short and long waiting times for surgery; a significant difference was observed between the two groups, with a significantly higher incidence of systemic recurrence in the long waiting time group; there was a significant difference between the two groups, with a significantly higher recurrence rate in the long waiting time group at 2 years postoperatively; asterisks indicate significant differences in intergroup comparisons

Figure 5A shows a comparison of the incidence rates of postoperative recurrence patterns for nonrecurrence, oligometastasis, and systemic recurrence between the short and long waiting time groups. A significant difference was observed between the two groups, with a higher incidence of systemic recurrence in the long waiting time group. Figure 5B shows a comparison of postoperative recurrence rates divided into 1-year intervals between the short and long waiting time groups. A significant difference was observed between the two groups, with a higher recurrence rate observed in the long waiting time group at 2–3 and 3–4 years postoperatively.

## Discussion

Although some observational studies have reported an association between waiting time from diagnosis to surgery and postoperative survival in patients with early esophageal adenocarcinoma, primarily in Caucasian patients undergoing upfront surgery,^10^ no study has focused solely on squamous cell carcinoma or on nonCaucasian patients with superficial esophageal cancer undergoing upfront surgery, and the acceptable surgical waiting time in these patients remains unclear.

Our study showed that even in clinical T1bN0 ESCC, primarily among Japanese patients undergoing upfront surgery, a surgical waiting time exceeding approximately 2 months was an independent prognostic factor for increased postoperative recurrence risk. Although cancer progresses over time, studies suggest that even relatively early stages, such as T1bN0, carry an increasing recurrence risk with time. In our study, when treatment was delayed, preoperative tumor staging was reevaluated, and the diagnosis of clinical T1bN0 was confirmed. Interestingly, no correlation was observed between pathological tumor depth, lymph node metastasis, and surgical waiting time. These findings suggest that the increased postoperative recurrence risk may reflect increased tumor malignancy not captured by conventional TNM classification. Although preliminary, the significantly higher rate of pathological supraclavicular lymph node metastasis in the long waiting time group suggests that prolonged waiting time may increase metastasis risk.

In advanced esophageal cancer, preoperative therapy is administered, and treatment response and adverse event-related changes in general condition may significantly influence outcomes. Several observational studies have examined the optimal interval between preoperative therapy and surgery in advanced disease.^11,12^ Consequently, studies evaluating surgical waiting time in advanced esophageal cancer involve strong confounding factors, likely explaining the lack of consensus. By contrast, T1bN0 esophageal cancer is treated with upfront surgery and is unaffected by preoperative therapy, making it suitable for evaluating the association between surgical waiting time and prognosis. cT1aN0 disease is generally treated endoscopically, such as with ESD, and even cT1bN0 cancer may initially undergo ESD in some cases. When radical esophagectomy is performed as additional treatment based on ESD pathology, prolonged surgical waiting time is unavoidable. In our study, prior ESD was significantly associated with longer waiting times; however, prior ESD was not associated with RFS. Although this differs from the hypothesis that prolonged waiting time increases recurrence risk, the prior ESD group represented only 12.5% (20 patients) of the cohort, limiting statistical power. Subgroup analysis showed that, among patients without prior ESD, similar to the overall cohort, shorter waiting times were associated with lower postoperative recurrence risk. Among the three patients with short waiting times after ESD, none developed recurrence. These findings suggest that even with ESD, shortening surgical waiting time may reduce postoperative recurrence risk. A retrospective study of patients undergoing ESD for cT1a-T1b esophageal cancer reported that waiting times up to 3 months before ESD did not affect long-term outcomes, although intrapapillary capillary loop changes—a predictor of tumor depth—were observed in some cases with prolonged waiting times. Because even superficial lesions eligible for ESD showed minimal tumor progression before ESD, ESD for cT1b esophageal cancer requires careful consideration of its indications.^13^ Clinically, surgical waiting time is partly determined by treatment sequence rather than tumor biology. Although prior ESD did not significantly affect postoperative survival in our analysis, the small number of patients means residual confounding cannot be excluded.

Although prolonged surgical waiting time was associated with increased postoperative recurrence risk, the 3-year CSS rate was higher in the long waiting time group than in the short waiting time group, although not significantly. Regarding recurrence patterns, the long waiting time group showed a high incidence of recurrence beyond 2 years postoperatively, with many systemic recurrences considered refractory. In contrast, the short waiting time group more frequently exhibited oligometastatic recurrence. Although recurrent esophageal cancer generally has a poor prognosis, several retrospective studies have shown that patients with “oligo (localized) recurrence” may achieve significantly improved post-recurrence survival when treated with local therapies such as surgical resection or radiation therapy.^14,15^ Despite the higher frequency of oligo-recurrence, the study did not demonstrate an association between CSS and surgical waiting time. Insufficient statistical power was considered a major factor explaining why no statistically significant association was demonstrated between surgical waiting time and CSS. With only nine esophageal cancer-related deaths, it was difficult to perform a sufficiently robust verification. The results regarding recurrence patterns were also interesting; however, they were not based on sufficient recurrence events and therefore represented exploratory findings. Given the limited number of esophageal cancer-related deaths (*n* = 9), these results should be interpreted with caution as exploratory findings. Additionally, recent improvements in treatments for recurrent esophageal cancer, including immune checkpoint inhibitors, may have rescued some patients, including those with systemic recurrence. Further validation in larger cohorts with sufficient events to evaluate CSS is warranted.

The National Comprehensive Cancer Network (NCCN) guidelines state that for patients who undergo upfront surgery and are found to have pathologic lymph node metastasis, adjuvant therapy may be considered.^1^ Meanwhile, in Japan, the Esophageal Cancer Practice Guidelines make no mention of adjuvant therapy for cT1bN0 esophageal cancer.^3^ Given these circumstances in Japan, the number of patients who received postoperative adjuvant therapy in the initial candidate patient group for this study was very small. Furthermore, this factor represented a major confounding variable with respect to its association with postoperative prognosis. Therefore, these patients were excluded from the final cohort under consideration. For patients with prolonged surgical waiting times who are suggested to be at high risk of recurrence, postoperative adjuvant therapy may be considered; however, it should be noted that the impact of postoperative adjuvant therapy on the results demonstrated in our study remains entirely unknown.

This study has some limitations. First, as this was a retrospective, multicenter observational study, careful consideration was required for potential biases, including preoperative diagnostic accuracy and quality control during surgery. Our analysis included patients who underwent systematic lymph node dissection and excluded those with fewer dissected lymph nodes. Regarding lymph node metastasis accuracy, 46 (28.8%) of 160 clinical N0 cases had pathologic lymph node metastases, and the 3-year RFS rate was 84.2% (95% CI 81.2–87.2%). In a prospective multicenter trial in Japan targeting cT1N0M0 esophageal cancer (Japan Clinical Oncology Group Study 0502; JCOG0502), the lymph node metastasis rate was 27.0%, and the 3-year RFS rate was 84.1% (95% CI 78.4–88.4%) in the surgery arm, which was nearly identical to our results.^16^ Therefore, although the time periods during which our study and the JCOG0502 study were conducted differed slightly, the quality of clinical lymph node diagnosis and surgery was considered to be assured. Regarding the accuracy of the clinical diagnosis of lymph node metastasis, the Japanese Classification of Esophageal Cancer, 12th Edition, describes the accuracy of clinical diagnosis of lymph node metastasis in patients with cT2–T4 tumors without neoadjuvant treatment using CT and PET-CT, targeting lymph nodes with a short-axis diameter of ≥ 5 mm. For CT, the negative predictive value (NPV) for upper mediastinal and abdominal lymph nodes at cutoff values of 5, 6, 7, and 8 mm in short axis was 69.6–74.7% and 50.6–59%, respectively. Likewise, the NPV for PET-CT was reported to be 94.4% for upper mediastinal lymph nodes and 92.4% for abdominal lymph nodes.^4^ The NPV for cN0 in our study was 71.3%, which we consider to represent generally acceptable accuracy. In esophageal cancer surgery, higher-volume institutions generally demonstrate lower perioperative mortality rates and a tendency toward improved long-term survival.^17^ Our results showed no differences in long-term outcomes between high-volume centers and city hospitals. Although no significant difference was observed between city hospitals and high-volume centers, city hospitals tended to have a higher proportion of patients with shorter waiting times. Therefore, we believe that bias resulting from differences in outcomes between institutions was not a concern. Second, the low statistical power was likely attributable to the relatively small number of recurrences and esophageal cancer-related death events. The AUC derived from ROC analysis for recurrence was 0.62, indicating low accuracy. Therefore, the cutoff value of 66.5 days established in our study should be regarded as an exploratory result rather than a definitive one. However, other cutoff values, particularly in the survival analysis at 2 months, generally showed trends regarding surgical waiting time and RFS. Therefore, the finding that our exploratory threshold of approximately 2 months of surgical waiting time does not increase the risk of recurrence was considered clinically acceptable. Although prolonged surgical waiting times were associated with an increased risk of recurrence, whether the approximately 2-month cutoff represents the optimal surgical waiting time requires confirmation in larger cohorts. Furthermore, sufficient statistical power for CSS analysis was difficult to achieve. Third, in this study, the definition of surgical waiting time necessarily differed between referred patients and patients diagnosed at the study institutions because the date of histological diagnosis at the previous institution could not always be fully verified. This heterogeneity introduced potential bias and may have obscured the biological interpretation of the 66.5-day cutoff value. Standardizing the baseline to a biologically meaningful starting point, such as the date of pathological confirmation prior to treatment, may eliminate this bias and enhance the interpretability of surgical waiting times.

In conclusion, this study demonstrated that delayed surgical waiting time in patients with cT1bN0M0 thoracic ESCC was associated with an increased risk of postoperative recurrence. This finding suggests that, in addition to baseline biological aggressiveness and occult nodal disease, disease progression over time, undetectable by conventional TNM classification, may increase recurrence risk. The optimal surgical waiting time is influenced by factors such as preoperative evaluations (including tumor and surgical feasibility assessment), adjustments to surgical schedules, and time required for patient decision-making. However, the threshold of approximately 2 months identified in this study is unlikely to increase postoperative recurrence risk. Using this as a guideline for determining surgical timing may help reduce recurrence risk. Ultimately, defining an acceptable surgical waiting time requires validation in larger cohorts.

## Supplementary Information

Below is the link to the electronic supplementary material.

Supplementary file1 (DOCX 301 kb)

## Data Availability

The datasets used or analyzed during the current study are available from the corresponding author on reasonable request.
