# Supplementary material for: Association between Surgical Waiting Time and Postoperative Recurrence Risk in Clinical T1bN0M0 Thoracic Esophageal Squamous Cell Carcinoma: A Multicenter Retrospective Observational Study
Source: Ann Surg Oncol. 2026 Jun 5;33(10):8910–21. doi: 10.1245/s10434-026-19840-6 (PMC13586040; doi:10.1245/s10434-026-19840-6)
Supplement: Supplementary file 1 [file 10434_2026_19840_MOESM1_ESM.docx]

Supplementary Figure 1:

From 2008 to 2021, 181 patients with cT1bN0 ESCC underwent upfront radical esophagectomy at seven participating institutions and were enrolled as candidates for this study. Of these, 160 patients who did not meet the exclusion criteria were included as eligible cases. Patients were stratified into a short waiting time group (120 cases) and a long waiting time group (40 cases) based on a cutoff value of 66.5 days for surgical waiting time determined by ROC analysis.

Supplementary Figure 2:

Histogram of the surgical waiting time, with a median time to surgery of 47.5 days (interquartile range, 34.25–66.75 days) and a standard deviation value of 36.6.

Supplementary Figure 3:

A. Kaplan–Meier curves for recurrence-free survival (RFS) in the subgroup without prior ESD. The long waiting time (>66.5 days) group showed significantly worse RFS than the short waiting time (<66.5 days) group (3-year RFS short/long, 87.6%/72.3%, *P*=0.003).

B. Kaplan–Meier curves for recurrence-free survival (RFS) in the subgroup with prior ESD. No significant differences were observed between the two groups in terms of RFS (3-year RFS short/long, 100%/75.3%, log-rank *P*=0.365).
